# Supplementary material for: Influenza A Virus Infection Reactivates Human Endogenous Retroviruses Associated with Modulation of Antiviral Immunity
Source: Viruses. 2022 Jul 21;14(7):1591. doi: 10.3390/v14071591 (PMC9320126; doi:10.3390/v14071591)
Supplement: Supplementary file 1 [file viruses-14-01591-s001.zip › MotifAnalysis.zip!/MotifOutput_SC35M/homerResults/motif3.similar.html]

motif3

## Information for motif3

A
C
G
T
A
T
G
C
A
C
G
T
A
C
T
G
A
C
T
G
A
C
G
T
A
T
C
G
C
A
T
G
A
C
T
G
A
C
T
G
G
T
C
A
A
G
T
C
  
Reverse Opposite:  

C
T
A
G
A
C
G
T
G
T
A
C
A
G
T
C
G
T
A
C
A
T
G
C
C
G
T
A
G
T
A
C
A
G
T
C
C
G
T
A
A
T
C
G
C
G
T
A
  

|  |  |
| --- | --- |
| p-value: | 1e-17 |
| log p-value: | -3.952e+01 |
| Information Content per bp: | 1.802 |
| Number of Target Sequences with motif | 11.0 |
| Percentage of Target Sequences with motif | 26.83% |
| Number of Background Sequences with motif | 179.7 |
| Percentage of Background Sequences with motif | 0.38% |
| Average Position of motif in Targets | 86.2 +/- 62.8bp |
| Average Position of motif in Background | 101.4 +/- 84.3bp |
| Strand Bias (log2 ratio + to - strand density) | 3.6 |
| Multiplicity (# of sites on avg that occur together) | 1.18 |
| Motif File: | file (matrix) reverse opposite |

### Similar de novo motifs found

|  |  |  |  |  |  |  |  |
| --- | --- | --- | --- | --- | --- | --- | --- |
| Rank | Match Score | Redundant Motif | P-value | log P-value | % of Targets | % of Background | Motif file |
| 1 | 0.602 | C G A T T G A C C A T G A C T G A T C G G A C T C A T G A C T G C T A G C A T G | 1e-11 | -25.722314 | 21.95% | 0.66% | motif file (matrix) |
